# Compressive Fatigue Investigation on High-Strength and Ultra-High-Strength Concrete within the SPP 2020

**DOI:** 10.3390/ma15113793

**Published:** 2022-05-26

**Authors:** Marco Basaldella, Marvin Jentsch, Nadja Oneschkow, Martin Markert, Ludger Lohaus

**Affiliations:** 1 Institute of Building Materials Science, Leibniz University Hannover, Appelstraße 9a, 30167 Hannover, Germany; m.jentsch@baustoff.uni-hannover.de (M.J.); n.oneschkow@baustoff.uni-hannover.de (N.O.); lohaus@baustoff.uni-hannover.de (L.L.); 2 Materials Testing Institute, University of Stuttgart, Pfaffenwaldring 4d, 70569 Stuttgart, Germany; martin.markert@mpa.uni-stuttgart.de

**Keywords:** high-strength concrete, ultra-high-strength concrete, compressive fatigue resistance, strain development, stiffness development

## Abstract

The influence of the compressive strength of concrete on fatigue resistance has not been investigated thoroughly and contradictory results can be found in the literature. To date, the focus of concrete fatigue research has been on the determination of the numbers of cycles to failure. Concerning the fatigue behaviour of high-strength concrete (HPC) and, especially, ultra-high-strength concrete (UHPC), which is described by damage indicators such as strain and stiffness development, little knowledge is available, as well as with respect to the underlying damage mechanisms. This lack of knowledge has led to uncertainties concerning the treatment of high-strength and ultra-high-strength concretes in the fatigue design rules. This paper aims to decrease the lack of knowledge concerning the fatigue behaviour of concrete compositions characterised by a very high strength. Within the priority programme SPP 2020, one HPC and one UHPC subjected to monotonically increasing and cyclic loading were investigated comparatively in terms of their numbers of cycles to failure, as well as the damage indicators strain and stiffness. The results show that the UHPC reaches a higher stiffness and a higher ultimate strain and strength than the HPC. The fatigue investigations reveal that the UHPC can resist a higher number of cycles to failure than the HPC and the damage indicators show an improved fatigue behaviour of the UHPC compared to the HPC.

## 1. Introduction

Developments in concrete technology nowadays allow the application of concrete compositions with ever-higher compressive strengths, which enable the construction of more filigree and slender structures. These structures are exposed to a higher extent to fatigue-relevant loads compared to massive structures due to their lower ratio of deadweight to non-static loads. For those structures, the fatigue resistance of the concrete becomes decisive for the design. At the same time, there has been a great demand in recent decades for types of structures for which fatigue-related stresses are characteristic, such as wind turbines or slender bridges made of high-strength or ultra-high-strength concrete. Thus, the research in recent decades has been more focused on the fatigue behaviour of concretes, e.g., [1,2,3,4].

The compressive fatigue resistance of plain concrete is described by the number of cycles to failure that the concrete can bear at a specified stress level [5] and expressed as so-called S/N-curves in standards and guidelines, e.g., [6,7]. For many decades, the influence of the concrete’s compressive strength on the fatigue resistance has been discussed in the literature, with controversial results and a consensus is still lacking. It should be mentioned that due to the typical scatter of fatigue test results and the resulting number of retry tests, the number of influences or, rather, variations of parameters in a special investigation is generally limited. Concurrently, the comparability between results of different investigations is often limited because of different fatigue loadings investigated and boundary conditions. With respect to the influence of compressive strength, the investigations in [8,9] showed that high-strength concretes resist lower numbers of cycles to failure than concrete compositions with lower compressive strengths. On the contrary, other investigations showed that the compressive strength has no influence on the fatigue resistance or that it is negligible [1,10,11]. In [12], even higher numbers of cycles to failure were determined for the higher strength concrete included. Overall, fewer investigations are documented in which concretes with different compressive strengths were investigated comparatively. As ultra-high-strength concretes came up recently, there are only a few fatigue investigations on plain ultra-high-strength concretes documented in the literature up to now [12,13,14]. 

**Figure 1:**
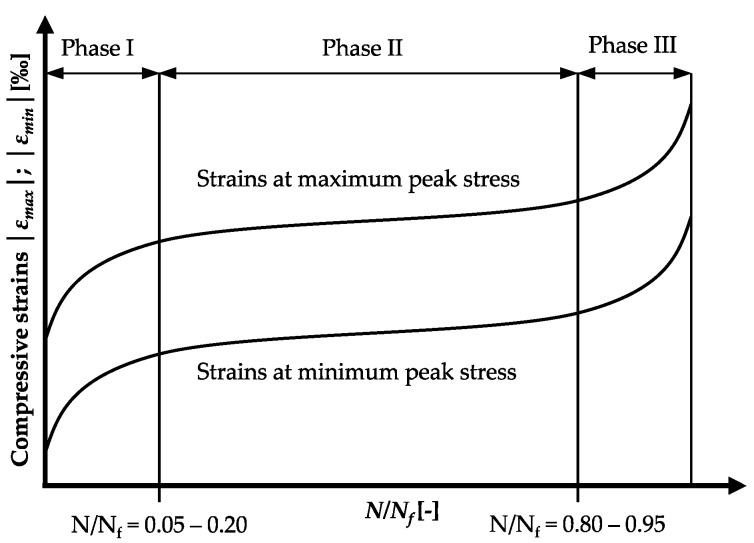
S-shaped strain developments during fatigue loading (schematic).

However, comparatively little research has been focussed on the concrete’s fatigue behaviour, especially on that of ultra-high-strength concrete, described by damage indicators, such as, e.g., the development of strain and stiffness in order to obtain more knowledge concerning the underlying damage mechanisms [1,2,3,15,16,17]. The developments of strain at the maximum and minimum peak stresses under compressive fatigue loading show typical s-shaped curves (Figure 1). For normal-strength concretes, the transitions from phase I to II and from II to III are located at about 5 to 20% and 80 to 95%, respectively, of the relative number of cycles to failure *N/N_f_* [1,3,4,18,19,20,21,22]. For high-strength concretes (HPC) and ultra-high-strength concretes (UHPC), phase I and III are generally shorter and less pronounced [8,11,13,23]. 

The stiffness of concrete within the fatigue process is usually described by the secant modulus in the decreasing branch of the hysteresis loop [15,19,22,24]. Similar to the developments of strain, the development of stiffness per load cycle shows an s-shaped curve. The evaluation of the results documented in the literature indicates that the reduction of stiffness until fatigue failure is lower for concretes with higher compressive strengths [16,19]. A comparison of the gradient of stiffness in phase II with respect to the numbers of cycles to failure *N_f_* in a double-logarithmic scale was conducted in [23], based on our own results and those documented in the literature [13,15,25] to evaluate possible differences between concretes with different concrete strengths. Here, differences were partially determined, but could not be clearly assigned to the influence of concrete strength due to possible influences of different testing laboratories. 

Overall, there is still a lack of knowledge concerning possible differences of the fatigue resistance and fatigue behaviour of high-strength and ultra-high-strength concrete, which leads to uncertainties concerning their treatment in the fatigue design rules. As a result, the currently valid design rules for compressive fatigue loading in the standards and guidelines, such as Eurocode 2 [7] or Model Code 2010 [6], consider concretes to be more sensitive to fatigue loading with increasing compressive strength [26,27,28]. Therefore, a strength-dependent reduction factor is included, which reduces the applicable fatigue resistance to an extent that can lead to an uneconomic usage of those concretes and even to a hindrance of the realisation of innovative concrete constructions.

Within the Priority Programme SPP 2020 ‘Cyclic Deterioration of High-Performance Concrete in an Experimental-Virtual Lab’, the fatigue behaviour and damage mechanisms of high-performance concretes under fatigue loading are investigated in the framework of 14 participating projects at different universities, each with a special focus in this research field. In order to ensure a certain level of comparability, one high-strength and one ultra-high-strength concrete composition are used as reference compositions in the investigations conducted by the different participating projects. Furthermore, the compressive fatigue behaviour of the reference high-strength and ultra-high-strength concrete are investigated comparatively by the so-called ‘central project’ for the purpose of a basic characterisation to be used as a reference in the different research projects. 

In this paper, the results of investigations in the compressive fatigue behaviour of the reference high-strength and ultra-high-strength concretes are presented comparatively. The numbers of cycles to failure and the damage indicators strain and stiffness are analysed with respect to the influence of the compressive strength. The fatigue investigations were carried out at two test laboratories in the framework of the SPP 2020 in order to superiorly evaluate the validity of the concrete-related differences identified in the number of cycles to failure and damage indicators.

However, since the fatigue behaviour of concrete cannot be considered detached from its behaviour under monotonically increasing loading, it is also presented and the fatigue behaviour is discussed considering the ’static’ material’s behaviour. The overall objective of the investigations presented here is to contribute to the decrease in the lack of knowledge and the previously described discussion in the literature concerning the influence of the compressive strength of concretes on their fatigue resistance.

## 2. Materials and Methods

### 2.1. Concrete Composition

The investigations were conducted on one high-strength concrete and one ultra-high-strength concrete, which are the reference concretes within the SPP 2020. The UHPC composition was developed based on the M3Q composition previously used in the SPP 1182 [29]. The composition of both reference concretes are given in Table 1.

**Figure A1:**
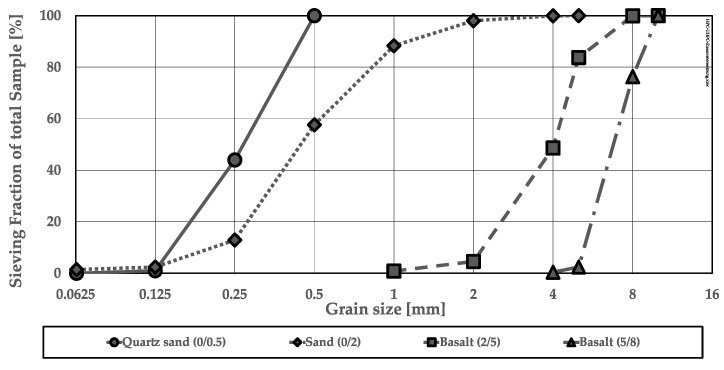
Grain size distribution of quartz sand, sand and basalt aggregate.

The grain size distributions of the quartz sand, sand and basalt aggregate are shown in Figure A1. The physical properties and the grain size distribution of the cement and the fine aggregates are shown in Table A1. The chemical properties of the cement are given in Table A2. Both superplasticisers used are on a PCE basis, while the stabiliser used modifies the viscosity.

As a complimentary investigation, the 28-day compressive strengths for the different batches produced were determined continuously by seven laboratories participating in the SPP 2020 since the start of the SPP 2020 in 2017. The tests of the cubic specimens were carried out in accordance with the guidelines given in DIN EN 12390-3:2019 [30]. The results were saved in a central database and a statistical analysis was performed. The edge length of the cubic specimens was either 100 or 150 mm. The storage conditions were either under water until testing according to DIN EN 12390-2:2009 [31] (marked as ‘wet’) or in standard climate conditions (20 °C/65% R.H.; marked as ‘dry’), due to the adaption of the lasted update to DIN EN 12390-2:2019 [32]. The number of results for each specimen size and loading condition are listed in Table A3 in the Appendix A. Due to the different sizes and storage conditions, a conversion of compressive strength according to DIN 1045-2:2008 [33] was taken into consideration. However, a general applicability of given conversion factors is questionable and, furthermore, they are not necessarily applicable for UHPC. Therefore, it was decided to consider the compressive strengths without conversion. 

**Figure A2:**
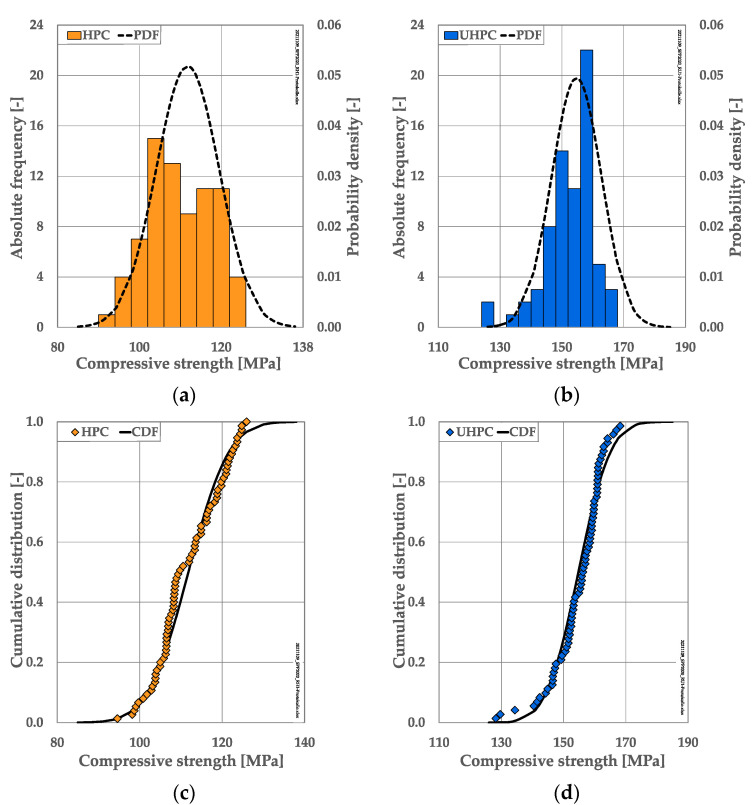
Absolute frequency and probability density for the (**a**) HPC and (**b**) UHPC. Cumulative distribution for the (**c**) HPC and (**d**) UHPC.

The frequency distribution and cumulative distribution of the compressive strength are shown in Figure A2 in the Appendix A. The compressive strength is approximately normally distributed for both concretes. The characteristic concrete compressive strength was calculated (Equation (1)) as 5%-fractile, in accordance with DIN EN 1990 [34].

(1) $$f_{ck}{=f}_{cm}-{\;k}_{n}\;\cdot {\;\sigma }_{x}\;\approx {\;f}_{cm}-\;1.64\;\cdot {\;\sigma }_{x}\;$$

where *f_ck_* is the characteristic compressive strength, *f_cm_* is the mean compressive strength, *k_n_* is the 5%-fractile factor according to [34] and *σ_x_* is the standard deviation. The mean compressive strength with its standard deviation and the characteristic compressive strength calculated are shown in Table 2 for both concrete types. 

Based on this analysis, the HPC was classified as C80/95 according to DIN EN 206:2021 [35] and the UHPC as C130/140 according to the draft of the DAfStb guideline [36]. It is possible that these classifications could be changed due to the inclusion of future results determined in the ongoing SPP 2020. It should be noted that the conversion of the compressive strengths according to [33] would not have led to different concrete strength classifications.

### 2.2. Specimens for the Fatigue Investigations

For the fatigue investigations, cylindrical specimens were prepared with a final height of *h* = 180 mm and a diameter of *d* = 60 mm. The HPC specimens were produced at the Institute of Building Materials Science, Leibniz University Hannover (IfB) and those of the UHPC were produced at the Institute of Concrete Structures, Technical University of Dresden (IMB). Two batches (HPC-a, HPC-b, respectively, UHPC-a, UHPC-b) were produced for each concrete. The production at those two laboratories was scheduled considering the expertise in the production of the respective concretes and the availability of the components of the concrete composition. 

All specimens were cast in cylindrical formworks with a height of about 250 mm, compacted using a vibrating table. The formwork was removed after 48 h and the specimens were stored under standard climate conditions (20 °C/65% R.H.) until testing. The specimens were prepared by sawing a few centimetres off the top and bottom to remove areas where the concrete may have been disturbed due to the production process. Additionally, the test surfaces of the specimens were ground parallel and polished to achieve a uniform stress distribution. The final height of the specimens ready to be tested was *h* = 180 mm. The test specimens were sent in shockproof boxes [37] to the testing laboratories.

### 2.3. Fatigue Test Programme and Experimental Set-Up 

The fatigue tests on both concretes were conducted under uniaxial compressive cyclic loading with constant maximum and minimum stress levels for each test. The minimum stress level was *S_min_* = 0.05 for all fatigue tests. The maximum stress levels were *S_max_* = 0.85 and *S_max_* = 0.75. The load frequency applied was *f_t_* = 1.0 Hz in all tests. The fatigue tests at *S_max_* = 0.75 were conducted at the IfB, while the fatigue tests at *S_max_* = 0.85 were conducted at the Materials Testing Institute, University Stuttgart (MPA). This procedure was chosen to achieve a comparability of the results between HPC and UHPC for each stress level (no influence of laboratory). Investigations at *S_max_* = 0.75 were additionally carried out at the MPA to investigate the stress level effect without laboratory influence. The number of fatigue tests conducted at each laboratory is summarised in Table 3, regarding concrete type, batch and stress level. The fatigue tests at the IfB were conducted at a specimen age between 79 and 97 days, whereas those at the MPA were conducted at a specimen age between 213 and 249 days, due to delays in the test scheduling.

The fatigue reference compressive strengths *f_cm,ref_* of the concrete specimens were tested just before conducting the fatigue investigations, using at least five specimens from the same batch and having the same geometry of the specimens used in the fatigue investigations (cylinder d/h = 60/180 mm). The tests were conducted force-controlled with a stress velocity of 0.5 MPa/s, using the same testing machine as for the fatigue tests. The resulting mean compressive strength of each concrete and batch was used as fatigue reference compressive strength *f_cm,ref_* for the fatigue tests to determine the compressive fatigue stresses required based on the stress level investigated (*S_min_ = σ_min_/f_cm,ref_; S_max_ = σ_max_/f_cm,ref_*).

**Figure 2:**
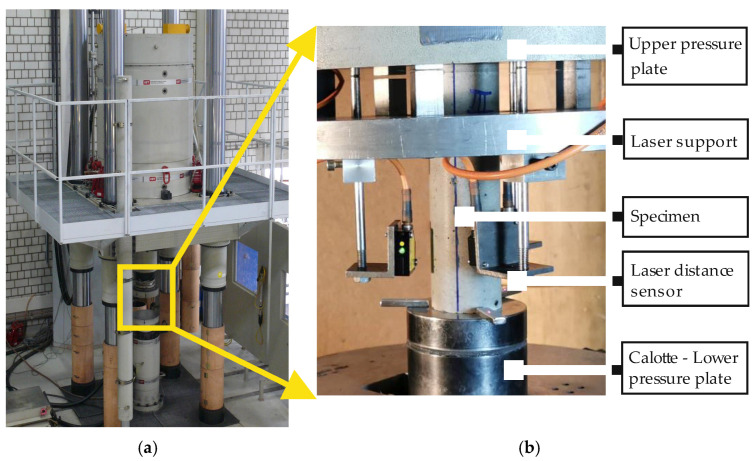
Test set-up at the IfB: (**a**) 1 MN servo-hydraulic actuator, (**b**) measurement set-up.

The fatigue tests were carried out using servo-hydraulic testing machines with 1 MN actuators, exemplarily shown in Figure 2a. The axial deformations were measured continuously in all tests using three laser distance sensors positioned on the circumference of the specimen at 0°, 120° and 240° (Figure 2b). The axial force and the displacement of the actuator were also recorded. In addition, the temperature on the specimen’s surface was measured at mid-height and 1 cm above/below the upper and lower pressure plates. Furthermore, the ambient temperature in the testing chamber was recorded. The fatigue failure occurred in a rather sudden and explosive way and, thus, crack pattern could not be analysed.

### 2.4. Analysis Methods

The fatigue resistance of the plain HPC and UHPC was analysed by evaluating the numbers of cycles to failure obtained from the tests. Statistical analyses were applied regarding the influence of the two batches of each concrete and the influence of the two concrete compositions on the numbers of cycles to failure. 

In addition, the maximum and minimum strains at peak stresses of the sinusoidal load curve and the stiffness were analysed as damage indicators. The maximum and minimum strains were obtained from the three laser distance sensors and averaged per specimen. In the analyses of the strains, the temperature increase due to cyclic loading was considered using a thermal expansion coefficient of α*_T_* = 1.0 × 10^−5^ K^−1^. This approach is considered sufficient due to the low temperature increase observed during the tests (max. 9 K for the HPC and 13 K for the UHPC). 

The total growths of maximum and minimum strain (*∆ε_max_*^0.0–1.0^*, ∆ε_min_*^0.0–1.0^) up to failure and the gradients of strain development in phase II (*grad ε_max_*^0.2–0.8^, *grad ε_min_*^0.2–0.8^) were analysed as parameters (cf. Table 4). Hereby, the gradients were determined between fixed values of *N/N_f_* = 0.20 and 0.80 from a linear regression analysis. 

The stiffness (*E_s_*) during the cyclic loading was calculated for each cycle as the secant modulus in the decreasing branch of the hysteresis loop. The gradient of the stiffness development in phase II (*grad E_s_*^0.2–0.8^) was determined corresponding to the approach for the gradient of strain. The reduction of stiffness up to failure was analysed as percentile value with respect to the initial stiffness at the beginning of the fatigue loading (*∆E_s_*^0.0–1.0^). 

The strain and stiffness developments were determined for each fatigue test, leading to a large number of individual curves. Therefore, averaged curves of the damage indicators were determined for each concrete batch and stress level for the graphical presentation. These curves were determined for identical test conditions by averaging the development of the individual damage indicator as a function of the relative number of load cycles, *N/N_f_*, and then multiplying this averaged curve by the mean value of the numbers of cycles to failure, *N_f_*. As a result of this procedure, the slopes of the curves are distorted. Hence, the depicted curves reflect the relative (higher/lower), but not the absolute relationships between the curves for the varying types of concretes investigated. Therefore, the mean values of the parameters, which were calculated based on the individual developments, are additionally given in Table A4 (Appendix A). They were used in the quantitative analyses.

## 3. Results

### 3.1. Stress–Strain Curves

**Figure 3:**
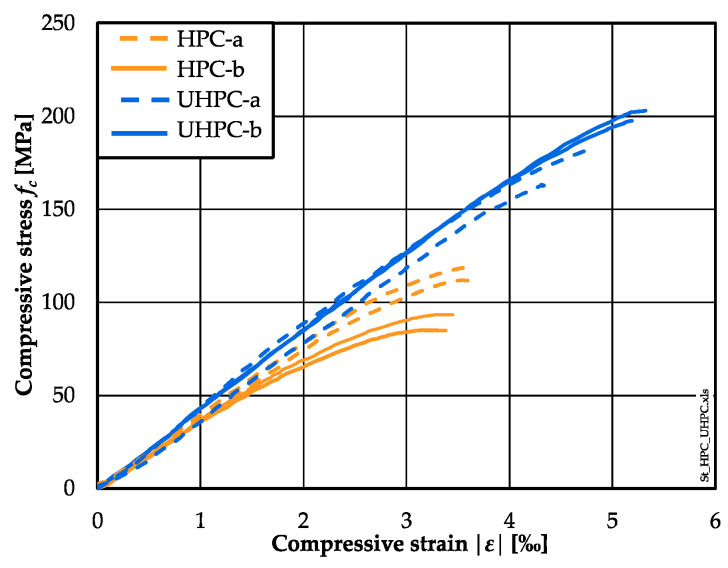
Stress–strain curves of HPC and UHPC per concrete batch (max. and min. curves).

Both concrete compositions were tested under monotonically increasing loading in order to obtain the fatigue reference compressive strength for the fatigue tests (cf. Section 2.3). The resulting stress–strain curves were additionally used to characterise their general material behaviour and to evaluate the differences in the fatigue test results with respect to the differences in the stress–strain curves. The enveloping stress–strain curves for each concrete batch are displayed in Figure 3. The stiffness was determined as the secant modulus of elasticity between 15% and 80% of the maximum stress. The mean values of the fatigue reference compressive strength *f_cm,ref_*, the ultimate strain *ε_cm_* (strain at maximum stress) and the stiffness *E*_0.15–0.80_ are summarised in Table 5.

It is noticeable from Figure 3 that the slopes of the stress–strain curves differ more strongly between the batches of the HPC compared to those of the UHPC. The differences in the slopes of the curves are smaller for the UHPC batches, but a considerable difference in the compressive strength is also visible. Furthermore, it can be seen that the non-linear part of the stress–strain curve of the HPC is more pronounced compared to that of the UHPC. It is clear from Table 5 that the stiffnesses of the HPC are smaller than those of the UHPC. The ultimate strains of the HPC are remarkably smaller than those of the UHPC. 

From Table 5 it can be seen that the fatigue reference compressive strength was determined as *f_cm,ref_ =* 116.2 MPa for batch HPC-a and *f_cm,ref_ =* 89.8 MPa for batch HPC-b, despite being of the higher concrete age. However, the 28-day cubic compressive strength of HPC-b was also significantly lower than that of HPC-a. Thus, this difference was batch-related and related to the scattering of the HPC (Figure A2). Moreover, the compressive reference strength of HPC-b is within the range of the results (min. *f_cm,ref_* = 82 MPa, max. *f_cm,ref_* = 117 MPa) determined in a previous round robin test [37]. The fatigue reference compressive strength of the UHPC was determined as *f_cm,ref_ =* 174.0 MPa for batch UHPC-a and *f_cm,ref_ =* 200.6 MPa for batch UHPC-b. The fatigue reference compressive strength of batch UHPC-a correlates with the results presented in [37]. Here, the higher compressive strength of UHPC-b might be due to the higher age of the specimens at testing. 

### 3.2. Numbers of Cycles to Failure

**Figure 4:**
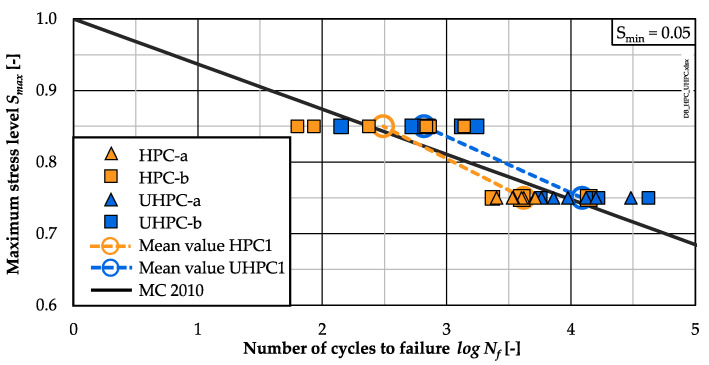
Number of cycles to failure for the HPC and UHPC.

The numbers of cycles to failure of the two concrete compositions are presented in Figure 4 as single and mean values. In addition, the S/N curve of Model Code 2010 [6] is included for the purpose of comparison. One batch was tested for each concrete at *S_max_* = 0.85, while specimens of both batches were investigated at *S_max_* = 0.75 (cf. Table 3). Therefore, the logarithmic numbers of cycles to failure at *S_max_* = 0.75 were analysed statistically for each concrete with the purpose of evaluating possibly significant differences between both batches. The probability values from the statistical test ANOVA were determined as *p*-value >> 0.05 for both concretes. Thus, no significant difference between the batches exists and the numbers of cycles to failure of HPC-a and HPC-b, respectively, UHPC-a and UHPC-b can be considered as belonging to the same group. Therefore, the mean number of cycles to failure of each concrete is calculated taking into account the single values of both batches at the lower stress level.

It can be seen from Figure 4 that the UHPC reached higher mean numbers of cycles to failure compared to the HPC at both stress levels and compared to the S/N curve of Model Code 2010. The mean number of cycles to failure of the HPC is slightly lower than the value given by the S/N curve at the lower stress level.

In detail, it is visible that most of the single values of the UHPC concrete at the lower stress level *S_max_* = 0.75 are higher than those of the HPC. From a statistical point of view, the influence of the concrete type on the mean values is significant at this stress level (ANOVA, probability value *p*-value << 0.05), which means a better fatigue resistance of the UHPC. The scattering of the single values of both concretes is higher at the stress level *S_max_* = 0.85 than that at the lower stress level *S_max_* = 0.75. In statistical terms, the influence of the concrete type is not significant (ANOVA, probability value *p*-value >> 0.05) for the higher stress level *S_max_ =* 0.85. 

### 3.3. Strain Development

**Figure 5:**
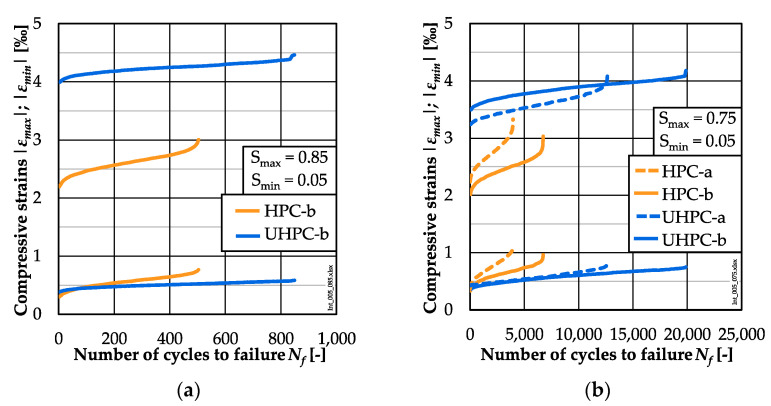
Averaged strain developments of HPC and UHPC at *S_max_* = 0.85 (**a**) and *S_max_* = 0.75 (**b**).

The averaged strain developments at the stress level *S_max_* = 0.85 are shown in Figure 5a and those at *S_max_* = 0.75 in Figure 5b for each concrete batch. It can be seen in Figure 5b that the strain developments at *S_max_* = 0.75 determined on specimens of different batches differed from each other, which is not surprising. When comparing the strain developments of the HPC and UHPC specimens at both stress levels, it can be seen that the initial maximum strains (*ε_max_*) and the following values of the UHPC were significantly higher than those of the HPC. Despite the same fatigue stress levels, the absolute stresses applied were higher for the UHPC specimens than for the HPC specimens due to their higher fatigue reference compressive strength (cf. Section 3.1). Thus, the higher maximum strains of the UHPC compared to the HPC can be traced back to the differences in the (monotonic) stress–strain curves, together with the higher fatigue reference compressive strength. 

The non-linear development of phase I and III of the UHPC is slightly shorter and the strain increase is less pronounced compared to the HPC, which corresponds to the trend of the results of [8,11,13,23]. In detail, the transitions between phase I and phase II, respectively, phase II and phase III were located at *N*/*N_f_* ≈ 0.17, respectively, *N/N_f_* ≈ 0.83 for the HPC and of *N/N_f_* ≈ 0.12, respectively, *N/N_f_* ≈ 0.88 for the UHPC.

The total growths of maximum and minimum strains (*∆ε_max_*^0.0–1.0^, *∆ε_min_*^0.0–1.0^) of the HPC were higher than those of the UHPC (cf. also Table A4). They increased with a decreasing maximum stress level for both concretes due to the higher numbers of cycles to failure. The HPC specimens showed a steeper gradient of strain in phase II, i.e., a higher strain increase per load cycle, than the UHPC ones at both stress levels (Table A4).

### 3.4. Stiffness Development

**Figure 6:**
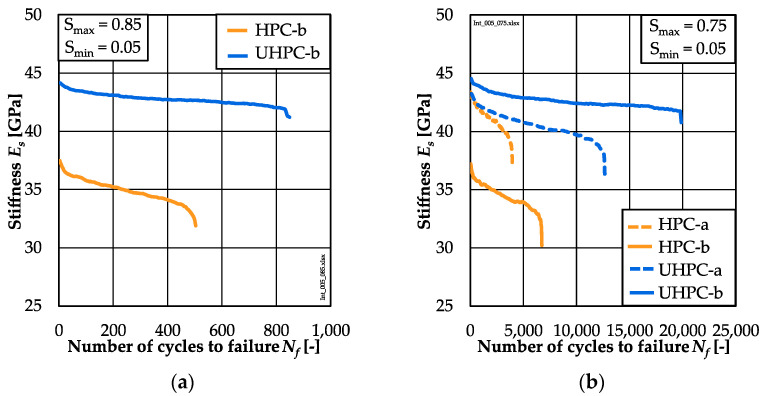
Averaged stiffness development of HPC and UHPC concretes for *S_max_* = 0.85 (**a**) and *S_max_* = 0.75 (**b**).

The averaged stiffness developments are shown in Figure 6a for *S_max_* = 0.85 and in Figure 6b for *S_max_* = 0.75. The initial stiffness and the following values of the UHPC are higher than those of the HPC. This is particularly evident for the concrete batches UHPC-b and HPC-b at both stress levels *S_max_* = 0.85 and *S_min_* = 0.75. A difference in the initial stiffness between the batches HPC-a and HPC-b can be noted at stress level *S_max_* = 0.75. These differences can be traced back to the respective stress–strain curves (non-linearity) and fatigue reference compressive strengths (cf. Section 3.1). The initial stiffness of the UHPC-a and UHPC-b batches are almost similar. 

The gradient of stiffness in phase II of the HPC is steeper than that of the UHPC at both stress levels and, thus, the same relationship exists as for the gradients of strain (cf. Section 3.3). It can be seen from Table A4 that the percentile reduction of stiffness (*∆Es*^0.0–1.0^*)* of the HPC is higher on average than that of the UHPC. This material dependency corresponds to results from [16]. Furthermore, the percentile reduction of stiffness of both concretes increases with the decreasing maximum stress level leading, respectively, to higher numbers of cycles to failure. 

## 4. Discussion

All tests were performed at two different laboratories with specimens stored and tested in the same conditions. Significant differences in the fatigue reference compressive strength *f_cm,ref_* of both batches of each concrete were determined (cf. Section 3.1). No significant difference concerning the mean numbers of cycles to failure of the two batches of each concrete could be determined at the lower stress level *S_max_* = 0.75 (cf. Section 3.2). Thus, the usage of the fatigue reference compressive strength for the determination of the fatigue stresses based on the stress levels equalised the batch influence with respect to the numbers of cycles to failure, although the tests were conducted at different laboratories. This was achieved by determining the fatigue reference compressive strength directly before carrying out the fatigue tests and by keeping the storage and testing conditions constant. 

Considering the influence of the concrete type, the mean number of cycles to failure was significantly higher for the UHPC at *S_max_* = 0.75 (cf. Section 3.2). Thus, the influence of the type of concrete was not equalised by the usage of the fatigue reference compressive strength at this stress level, contrary to the batch influence. A higher mean number of cycles to failure of the UHPC was also found at the higher stress level S_max_ = 0.85, but the difference was not significant. Altogether, the differences in the number of cycles to failure can be reliably related to the different materials’ fatigue behaviour based on the previous considerations.

**Figure 7:**
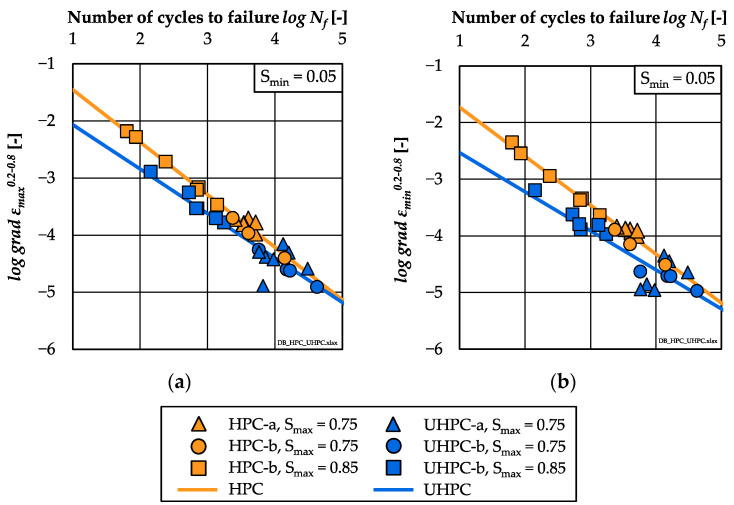
Log. gradients of strain in phase II related to log. numbers of cycles to failure of HPC and UHPC; (**a**) gradients of maximum strain and (**b**) gradients of minimum strain.

In addition to the numbers of cycles to failure, the damage indicators strain and stiffness were investigated comparatively for the HPC and UHPC. The analyses of the strain developments showed that the HPC exhibited a higher total growth of the maximum and minimum strain than the UHPC, although fewer numbers of cycles were suffered until failure. This correlates to the steeper gradient of strain in phase II of the HPC (cf. Section 3.3). In Figure 7a,b, the single values of the gradients of the maximum and minimum strain in phase II (*grad ε_max_*^0.2–0.8^, *grad ε_min_*^0.2–0.8^) are displayed with respect to the numbers of cycles to failure in double-logarithmic graphs.

Linear relations between the logarithmic gradients of, respectively, the maximum and minimum strain and the logarithmic numbers of cycles to failure were found for both concretes. The regression lines can be expressed by the following equations (Equations (2)–(5)):
HPC:$\log N_{f}=-0.921\;\cdot \log\left({\mathrm{grad}\;\varepsilon }_{\max}^{0.2–0.8}\right)-\;0.532$ R² = 0.98(2)HPC:$\log N_{f}=-0.863\;\cdot \log\left({\mathrm{grad}\;\varepsilon }_{\min}^{0.2–0.8}\right)-\;0.870$ R² = 0.98(3)UHPC:$\log N_{f}=-0.778\;\cdot \log\left({\mathrm{grad}\;\varepsilon }_{\max}^{0.2–0.8}\right)-\;1.290$ R² = 0.88(4)UHPC:$\log N_{f}=-0.689\;\cdot \log\left({\mathrm{grad}\;\varepsilon }_{\min}^{0.2–0.8}\right)-\;1.844$ R² = 0.80(5)

The gradients of strain decrease with decreasing stress levels. Furthermore, the gradients of the minimum strain of both concretes are flatter than the gradients of the maximum strain. Both findings confirm the results of [2]. Furthermore, the regression lines of the UHPC are located below the regression lines of the HPC, which corresponds to the findings in [13,15,16]. This means that smaller gradients of strain or, rather, increases of strain per load cycle are reached by the UHPC for the same number of cycles to failure.

**Figure 8:**
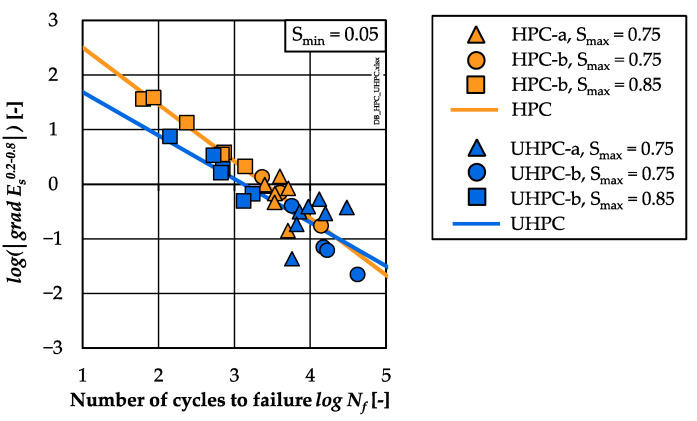
Log. gradients of stiffness in phase II (*log(│grad* *E_s_*^0.2–0.8^*│*) related to log. numbers of cycles to failure of HPC and UHPC.

The analyses of the stiffness developments showed a higher percentile reduction of stiffness of the HPC compared to the UHPC, although fewer numbers of cycles were suffered until failure (cf. Section 3.4). Similar to the strain development, the gradient of stiffness in phase II (*grad E_s_*^0.2–0.8^) of the HPC was steeper than that of the UHPC. In Figure 8, the single values of the gradients of stiffness are presented comparatively with respect to the numbers of cycles to failure in double-logarithmic graphs. Linear relations between the logarithmic gradients of stiffness and the logarithmic numbers of cycles to failure were found for both concretes (Equations (6) and (7)), the same as for the gradients of strain:
HPC:${\log N}_{f}=-1.041\;\cdot \log\left(\;\left|{\;\mathrm{grad}\;E}_{s}^{0.2–0.8}\right|\;\right)+3.545$ R² = 0.93(6)UHPC:${\log N}_{f}=-0.796\;\cdot \log\left(\;\left|{\;\mathrm{grad}\;E}_{s}^{0.2–0.8}\right|\;\right)+2.484$ R² = 0.69(7)

It is visible that the gradients of stiffness decrease with decreasing stress levels, which corresponds to results of [2,15,17]. It is noticeable that the results of the UHPC specimens of batch UHPC-a show a larger scattering (stress level *S_max_* = 0.75). Here, both regressions lines cross each other. In the range of lower *log N_f_* ≈ 4, the regression line of the UHPC is located below that of the HPC. Thus, flatter gradients of stiffness or, rather, a lower stiffness reduction per load cycle are observable for the UHPC compared to the HPC for the same number of cycles to failure, which correlates to the findings from [16]. Without the high scattering of batch UHPC-a, this statement might have been drawn also for *log N_f_* > 4. The differences between the regression lines with respect to gradients of strain and stiffness in phase II (Figure 7 and Figure 8) reveal a different material-dependent fatigue behaviour of both concretes.

According to [8,9] and to the design approach of standards and guidelines (e.g., [6,7]), a lower fatigue resistance of the UHPC compared to the HPC was expected. However, the UHPC investigated showed a higher fatigue resistance than the HPC. Thus, the results confirm observations documented in [12]. However, the fatigue tests conducted in the investigation presented in this paper were limited and, thus, further investigations are necessary to enable a broader view.

## 5. Conclusions

The compressive fatigue resistance of a high-strength concrete and an ultra-high-strength concrete, which are the reference concretes in the Priority Programme SPP 2020, were investigated comparatively considering the numbers of cycles to failure and the damage indicators strain and stiffness. The main objective of this study was to contribute to a decrease in the lack of knowledge concerning the influence of compressive strength on the fatigue resistance of concretes. The fatigue investigations were conducted at two stress levels, *S_max_ =* 0.85 and 0.75, with the same minimum stress level, *S_min_* = 0.05. The loading frequency was kept constant at *f_t_* = 1.0 Hz. The fatigue results were also discussed with respect to the stress–strain curves due to monotonically increasing loading. The experimental investigations were conducted at two laboratories for the purpose of the evaluation of the validity of the concrete-related differences identified in the numbers of cycles to failure and damage indicators, thus reaching more reliable conclusions. The main findings can be summarised as follows:

- The UHPC reached higher mean numbers of cycles to failure than the HPC at both stress levels investigated. Furthermore, the difference in mean numbers of cycles to failure was statistically significant (ANOVA, *p*-value << 0.05) at the lower level. Thus, a negative influence of the higher compressive strength of the UHPC on the numbers of cycles to failure was not observed in the investigations presented.
- The damage indicators showed a smaller total growth of strains and a smaller percentile reduction of stiffness of the UHPC compared to the HPC. Furthermore, the gradients of strain and stiffness in phase II, i.e., the increase in strain and decrease in stiffness per load cycle, respectively, of the UHPC were smaller than those of the HPC. In summary, the UHPC showed a less pronounced damage evolution compared to the HPC. Furthermore, the damage indicators reveal a different material-dependent fatigue behaviour.
- A batch influence on the results of the reference compressive strength for both concretes was identified. For each concrete, this batch influence was not found in the numbers of cycles to failure. Thus, the batch influence was equalised due to the determination of the tested fatigue stresses, based on the respective reference compressive strength of the batch.

Overall, a higher fatigue sensitivity could not be found for the UHPC compared to the HPC investigated. Thus, this result contradicts the results from [8,9] and confirms the results of [12]. It also contradicts the current approach of the design standards and guidelines [6,7], which consider the compressive fatigue sensitivity of concretes to increase with the increasing compressive strength. The observed trend here should be systematically investigated on a broader database of results. 

## Figures and Tables

**Table 1:** Compositions of concretes.

Component	Unit	HPC	UHPC
CEM I 52.5 R-HS/NA (Holcim Sulfo, Lägerdorf, Germany)	[kg/m³]	500	795
Silica fume (Sika^®^ Silicoll P)	[kg/m³]	-	169
Quartz powder (Quarzwerke MILLSIL^®^ W12, Frechen, Germany)	[kg/m³]	-	198
Quartz sand (0/0.5 mm) (Quarzwerke H33, Haltern, Germany)	[kg/m³]	75	971
Sand (0/2 mm) (Tündern, Germany)	[kg/m³]	850	-
Basalt (2/5 mm) (Ölberg, Germany)	[kg/m³]	350	-
Basalt (5/8 mm) (Ölberg, Germany)	[kg/m³]	570	-
Superplasticiser (BASF MasterGlenium^®^ ACE 460, Germany)	[kg/m³]	5	-
Superplasticiser (BASF MasterGlenium^®^ ACE 394, Germany)	[kg/m³]	-	24
Stabiliser (BASF MasterMatrix^®^ SDC 100, Germany)	[kg/m³]	2.85	-
Water	[kg/m³]	176	188
w/c ratio; w/c_eq_ ratio	[-]	0.35	0.19

**Table 2:** Classification of concretes based on tests of seven SPP 2020 laboratories.

Concrete	Qty	*f_cm_*	*SD*	*f_ck_*	Classification
	[-]	[MPa]	[MPa]	[MPa]	
HPC	75	111.8	7.7	99.2	C80/95
UHPC	72	154.8	8.1	141.5	C130/140

**Table 3:** Number of fatigue tests conducted.

Concrete	HPC-a	UHPC-a	HPC-b	UHPC-b
***S_min_***/***S_max_***	IfB	IfB	MPA	MPA
0.05/0.75	7	7	3	4
0.05/0.85	-	-	6	6

**Table 4:** Overview of parameters used.

Parameter	Unit	Description
$\sigma_{\max}\;;{\;\sigma }_{min}$	[MPa]	Maximum or minimum peak stress
$\varepsilon_{\max}{\;;\;\varepsilon }_{min}$	[‰]	Strain at maximum or minimum peak stress
$\Delta \varepsilon_{\max}^{0.0–1.0}{=\varepsilon }_{\max}^{1.0}-{\;\varepsilon }_{\max}^{0.0}$	[‰]	Total growth of strain at maximum stress, *N/N_f_* = 0.0–1.0
$\Delta \varepsilon_{min}^{0.0–1.0}=\varepsilon_{min}^{1.0}-\varepsilon_{min}^{0.0}$	[‰]	Total growth of strain at minimum stress, *N/N_f_* = 0.0–1.0
${\mathrm{grad}\;\varepsilon }_{\max}^{0.2–0.8}$	[-]	Gradient of maximum strain development in phase II (*N/N_f_* = 0.2–0.8)
${\max\;\varepsilon }_{min}^{0.2–0.8}$	[-]	Gradient of minimum strain development in phase II (*N/N_f_* = 0.2–0.8)
$E_{s}=\frac{\sigma_{\max}-{\;\sigma }_{min}}{\varepsilon_{\max}-{\;\varepsilon }_{min}}$	[MPa]	Stiffness due fatigue loading
$\Delta E_{s}^{0.0–1.0}=\frac{E_{s}^{0.0}-{\;E}_{s}^{1.0}}{E_{s}^{0.0}}$	[%]	Percentile reduction of stiffness, *N/N_f_* = 0.0–1.0
${\max\;E}_{s}^{0.2–0.8}$	[MPa]	Gradient of stiffness development in phase II (*N/N_f_* = 0.2–0.8)

**Table 5:** Mean values of the fatigue reference compressive strength, ultimate strain and stiffness.

Concrete	Qty	Age	*f_cm,ref_*	*SD*	*ε_cm_*	*E*_0.15–0.80_
	[-]	[d]	[MPa]	[‰]	[MPa]	[MPa]
HPC-a	6	79	116.2	2.2	−3.67	36,700
HPC-b	4	213	89.8	3.0	−3.31	33,200
UHPC-a	6	97	174.0	5.6	−4.57	41,200
UHPC-b	5	249	200.6	1.9	−5.24	41,700

## Data Availability

Data available on request.

## Appendix A

**Table A1:** Physical properties and grain size distribution of cement and fine aggregates.

Materials	Density	Fineness	d_10_	d_50_	d_90_
	[kg/dm³]	[cm²/g]	[µm]		
CEM I 52.5 R-HS/NA	3.18	3969	1.60	11.25	33.18
Silica fume	2.23	-	7.01	17.37	32.27
Quartz powder	2.66	-	2.09	12.71	37.18

**Table A2:** Chemical properties of cement CEM I 52.5 R-HS/NA.

Materials	Chemical Composition [%]					
	SiO_2_	Al_2_O_3_	Fe_2_O_3_	CaO	MgO	SO_3_
CEM I 52.5 R-HS/NA	21.41	3.97	4.79	65.41	0.85	2.98

**Table A3:** Number of cubic compressive strength test results analysed for the characterisation.

Concrete	Wet ^1^	Wet ^1^	Dry ^2^	Dry ^2^	Total
	150 mm	100 mm	150 mm	100 mm	
HPC	16	38	3	18	**75**
UHPC	5	6	0	61	**72**

^1^ Storage conditions according to DIN EN 12390-3:2009—stored under water until testing. ^2^ Storage conditions according to DIN EN 12390-3:2019—stored under water for 6 days and then under standard climate conditions until testing.

**Table A4:** Mean values of parameters of fatigue damage indicators investigated.

Concrete			HPC-a	HPC-b	UHPC-a	UHPC-b
0.85/0.05	*∆ε_max_* ^0.0–1.0^	[‰]	-	0.87	-	0.52
	*∆ε_min_* ^0.0–1.0^	[‰]	-	0.50	-	0.22
	${\mathrm{grad}\;\varepsilon }_{\max}^{0.2–0.8}$	[-]	-	2.57 × 10^−3^	-	4.67 × 10^−5^
	${\mathrm{grad}\;\varepsilon }_{\min}^{0.2–0.8}$	[-]	-	1.59 × 10^−3^	-	2.38 × 10^−5^
	*∆E_s_* ^0.0–1.0^	[%]	-	15.97	-	7.36
	${\mathrm{grad}\;E}_{s}^{0.2–0.8}$	[MPa]	-	−16.38	-	−2.61
**Concrete**			**HPC-a**	**HPC-b**	**UHPC-a**	**UHPC-b**
0.75/0.05	*∆ε_max_* ^0.0–1.0^	[‰]	1.21	1.17	0.99	0.78
	*∆ε_min_* ^0.0–1.0^	[‰]	0.81	0.71	0.41	0.44
	${\mathrm{grad}\;\varepsilon }_{\max}^{0.2–0.8}$	[-]	1.25 × 10^−4^	1.16 × 10^−4^	4.10 × 10^−5^	2.92 × 10^−5^
	${\mathrm{grad}\;\varepsilon }_{\min}^{0.2–0.8}$	[-]	1.63 × 10^−4^	7.66 × 10^−5^	2.31 × 10^−5^	1.82 × 10^−5^
	*∆E_s_* ^0.0–1.0^	[%]	16.62	21.80	16.08	9.52
	${\mathrm{grad}\;E}_{s}^{0.2–0.8}$	[MPa]	−0.78	−0.75	−0.30	−0.14

## References

[1] Hohberg R. (2004). Zum Ermüdungsverhalten von Beton. On the Fatigue Behaviour of Concrete. Ph.D. Thesis.

[2] Oneschkow N. (2016). Fatigue behaviour of high-strength concrete with respect to strain and stiffness. Int. J. Fatigue.

[3] Thiele M. (2016). Experimentelle Untersuchungen und Analyse der Schädigungsevolution in Beton unter Hochzyklischen Ermüdungsbeanspruchungen. Experimental Investigation and Analysis of the Damage Development in Concrete Subjected to High-Cycle Fatigue. Ph.D. Thesis.

[4] Viswanath S., LaFave J.M., Kuchma D.A. (2021). Concrete compressive strain behaviour and magnitudes under uniaxial fatigue loading. Constr. Build. Mater..

[5] Comité Euro-international du Beton (CEB) (1988). Fatigue of Concrete Structures—State of the Art Report. Bull. Dinf..

[6] Fédération Internationale du Béton (2013). Fib Model Code for Concrete Structures 2010.

[7] (2010). Eurocode 2: Design of Concrete Structures—Part 2: Concrete Bridges—Design and Detailing Rules.

[8] Kim J.K., Kim Y.Y. (1996). Experimental study of the fatigue behavior of high strength concrete. Cem. Concr. Res..

[9] Zhao G.Y., Wu P.G., Bai L.M. Research on fatigue behavior of high-strength concrete under compressive cyclic loading. In Proceeding of the 4th International Symposium on Utilization of High-strength/High-performance concrete.

[10] Bennett E.W., Muir S.E.S.J. (1967). Some fatigue tests of high-strength concrete in axial compression. Mag. Concr. Res..

[11] Petković G., Rosseland S., Stemland H. (1992). High Strength Concrete SP3—Fatigue. Report 3.2: Fatigue of High Strength Concrete.

[12] Kono S., Hasegawa H., Mori K., Ichioka Y., Sakashita M., Watanabe F. Low cycle fatigue characteristics of high strength concrete. Proceedings of the 8th International Symposium on Utilization of High-Strength and High-Performance Concrete.

[13] Wefer M. (2010). Materialverhalten und Bemessungswerte von Ultrahochfestem Beton unter Einaxialer Ermüdungsbeanspruchung. Material Behaviour and Design Values of Ultra-High-Strength Concrete Subjected to Uniaxial Fatigue Loading. Ph.D. Thesis.

[14] Deutscher M., Tran N.L., Scheerer S. (2019). Experimental Investigations on the Temperature Increase of Ultra-High Performance Concrete under Fatigue Loading. Appl. Sci..

[15] Petković G. (1991). Properties of Concrete Related to Fatigue Damage with Emphasis on High Strength Concrete. Ph.D. Thesis.

[16] Do M.T., Chaallal O., Aïtcin P.C. (1993). Fatigue Behavior of High-Performance Concrete. J. Mater. Civ. Eng..

[17] Oneschkow N., Timmermann T. (2022). Influence of the composition of high-strength concrete and mortar on the compressive fatigue behaviour. Mat. Struct..

[18] Klausen D. (1978). Festigkeit und Schädigung von Beton bei Häufig Wiederholter Beanspruchung. Strength and Damage of Concrete Subjected to Often Repeated Loading. Ph.D. Thesis.

[19] Holmen J.O. (1982). Fatigue of Concrete by Constant and Variable Amplitude loading. ACI Symposium Publication.

[20] Dyduch K., Szerszen M., Destrebecq J.-F. (1994). Experimental investigation of the fatigue strength of plain concrete under high compressive loading. Mat. Struct..

[21] Hümme J., von der Haar C., Lohaus L., Marx S. (2016). Fatigue behaviour of a normal-strength concrete—Number of cycles to failure and strain development. Struct. Concr..

[22] Isojeh B., El-Zeghayar M., Vecchio F.J. (2017). Concrete Damage under Fatigue Loading in Uniaxial Compression. ACI Mater. J..

[23] Oneschkow N. (2014). Analyse des Ermüdungsverhaltens von Beton anhand der Dehnungsentwicklung. Analysis of the Fatigue Behaviour of Concrete with Respect to the Development of Strain. Ph.D. Thesis.

[24] Scheiden T., Oneschkow N. (2019). Influence of coarse aggregate type on the damage mechanism in high-strength concrete under compressive fatigue loading. Struct. Concr..

[25] Sparks P.R. (1982). The Influence of Rate of Loading and Material Variability on the Fatigue Characteristics of Concrete. Int. Concr. Abstr. Portal.

[26] König G., Danielewicz I. (1994). Ermüdungsfestigkeit von Stahlbeton- und Spannbetonbauteilen mit Erläuterungen zu den Nachweisen gemäß CEB-FIP Model Code 1990; Fatigue Strength of Reinforced and Prestressed Concrete Components with Explanations of the Verifications According to CEB-FIP Model Code 1990.

[27] Oneschkow N., Lohaus L. (2017). Zum Ermüdungsnachweis von druckschwellbeanspruchtem Beton, Teil 1 About the design concept for compressive fatigue loading of concrete—Structure of the fatigue design concept. Beton Stahlbetonbau.

[28] Oneschkow N., Lohaus L. (2017). Zum Ermüdungsnachweis von druckschwellbeanspruchtem Beton, Teil 2; About the design concept for compressive fatigue loading of concrete—Safety considerations and potential of further developments. Beton Stahlbetonbau.

[29] Schmidt M., Fehling E., Fröhlich S., Thiemicke J. (2014). Sustainable Building with Ultra-High Performance Concrete—Results of the German Priority Programme 1182 funded by Deutsche Forschungsgemeinschaft (DFG). Structural Materials and Engineering Series.

[30] (2019). Testing Hardened Concrete—Part 3: Compressive Strength of Test Specimens.

[31] (2009). Testing Hardened Concrete—Part 2: Making and Curing Specimens for Strength Tests.

[32] (2019). Testing Hardened Concrete—Part 2: Making and Curing Specimens for Strength Tests.

[33] (2008). Concrete, Reinforced and Prestressed Concrete Structures—Part 2: Concrete—Specification, Properties, Production and Conformity—Application rules for DIN EN 206-1.

[34] (2010). Eurocode 0: Basis of Structural Design; German Version EN 1990-2002 + A1:2005 + A1:2005/AC:2010.

[35] (2021). Concrete—Specification, Performance, Production and Conformity; German Version EN 206:2013+A2:2021.

[36] Deutscher Ausschuss für Stahlbeton [German Committee for Reinforced Concrete] (2019). DAfStb-Richtlinie Ultrahochfester Beton, Teil 1 Bemessung und Konstruktion; DAfStb Guideline for Ultra-High-Strength Concrete, Part 1 Design and Construction.

[37] Basaldella M., Oneschkow N., Lohaus L. (2021). Influence of the specimen production and preparation on the compressive strength and the fatigue resistance of HPC and UHPC. Mater. Struct..

